# Rapid identification and drug resistance screening of respiratory pathogens based on single-cell Raman spectroscopy

**DOI:** 10.3389/fmicb.2023.1065173

**Published:** 2023-01-26

**Authors:** Ziyu Liu, Ying Xue, Chun Yang, Bei Li, Ying Zhang

**Affiliations:** ^1^Department of Pediatric Respiratory, The First Hospital of Jilin University, Changchun, China; ^2^School of Life Science, Jilin University, Changchun, China; ^3^HOOKE Instruments Ltd., Changchun, China; ^4^Department of Laboratory Medicine, First Hospital of Jilin University, Changchun, Jilin, China; ^5^The State Key Lab of Applied Optics, Changchun Institute of Optics, Fine Mechanics and Physics, Chinese Academy of Sciences (CAS), Changchun, China

**Keywords:** drug resistance, respiratory pathogens, single-cell, Raman spectroscopy, rapid identification

## Abstract

Respiratory infections rank fourth in the global economic burden of disease. Lower respiratory tract infections are the leading cause of death in low-income countries. The rapid identification of pathogens causing lower respiratory tract infections to help guide the use of antibiotics can reduce the mortality of patients with lower respiratory tract infections. Single-cell Raman spectroscopy is a “whole biological fingerprint” technique that can be used to identify microbial samples. It has the advantages of no marking and fast and non-destructive testing. In this study, single-cell Raman spectroscopy was used to collect spectral data of six respiratory tract pathogen isolates. The T-distributed stochastic neighbor embedding (t-SNE) isolation analysis algorithm was used to compare the differences between the six respiratory tract pathogens. The eXtreme Gradient Boosting (XGBoost) algorithm was used to establish a Raman phenotype database model. The classification accuracy of the isolated samples was 93–100%, and the classification accuracy of the clinical samples was more than 80%. Combined with heavy water labeling technology, the drug resistance of respiratory tract pathogens was determined. The study showed that single-cell Raman spectroscopy–D_2_O (SCRS–D_2_O) labeling could rapidly identify the drug resistance of respiratory tract pathogens within 2 h.

## Graphical Abstract

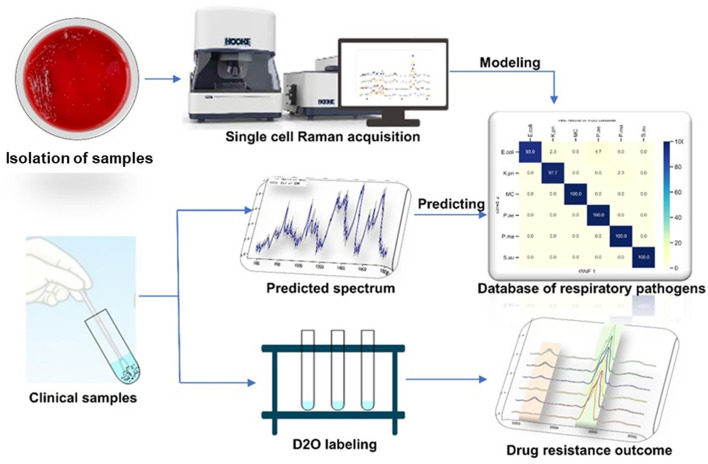

## Introduction

Antibiotic resistance to bacterial pathogens has been found in every country across the globe, and everyone can take action against it (Hernando-Amado et al., 2020). Meanwhile, the administration of broad-spectrum antibiotics in patients leads to the emergence of multidrug-resistant bacteria, posing a considerable risk to human health and life (Hay et al., 2018). To date, many assays used to identify respiratory viral and atypical bacterial methods have been developed such as the FilmArray Blood Culture Identification (BCID) panel (BioFire Diagnostics, LLC.), the Verigene Gram-positive blood culture (BC-GP), and Gram-negative blood culture (BC-GN). They are commercially available (Ramanan et al., 2018), while there are limited methods used for typical respiratory bacteria testing, especially combined with antibiotic-sensitive assays. Respiratory infections have been identified as a leading cause of morbidity and mortality worldwide (Prats et al., 2002), and there is an urgent need to develop rapid bacterial tests and diagnostic methods for antibiotic-resistant strains to address this global challenge. Therefore, timely treatment with appropriate antibiotic therapy can lead to better clinical outcomes and can decrease the generation of resistant strains (Prats et al., 2002).

The mechanisms of antibiotic resistance in strains mainly include enzyme production, which can destroy antibiotics, antibiotic target modulation, avoidance of existence through the cell membrane, and the initiative efflux system to the antibiotics (Blair et al., 2015; Peterson and Kaur, 2018). These mechanisms use several methods in sensitivity assays, such as broth microdilution, the microdilution test, the agar diffusion test, and the automated susceptibility testing systems (Verma et al., 2021). These conventional methods are time-consuming, while clinicians choose drugs based on experience that may lead to more drug-resistant bacteria. The commercial method for the rapid antibiotic resistance test usually depends on the PCR-based amplification reaction to identify the abnormally existing bases. At the same time, it cannot provide information about antibiotic susceptibilities (Burnham et al., 2017). Thus, developing a rapid testing system is essential to integrate confirmation of the antibiotic-resistant strains and information on sensitive antibiotics.

Raman spectroscopy can quickly and non-destructively detect microbial cell chemical components (Kanno et al., 2021). Raman spectra of individual cells contain information on nucleic acids, proteins, carbohydrates, lipids, and pigments, which can characterize the genotype, phenotype, and physiological state of microorganisms. Single-cell Raman spectroscopy (SCRS) is a “whole biological fingerprint” technique that can be used to identify microbial samples (Wang et al., 2021). It can be used for *in situ*, non-invasive, and non-labeled detection of samples, and has a great application value for qualitative analysis, quantitative analysis, and molecular structure determination (Bergholt et al., 2017). Deuterium (D) in heavy water can label cellular biomolecules through non-enzyme-catalyzed H/D exchange and enzyme-catalyzed integration equilibrium. The deuterium in the water was transferred into the substance by the reduction process of coenzyme I and coenzyme II in the bacteria cultured in heavy water, which shifted the C–H peak (2,800–3,000 cm^−1^) in the Raman spectrum of bacteria, and the C–D peak (2,000–2,300 cm^−1^) appeared, which became a biomarker of bacterial metabolic activity at the single-cell level (Song et al., 2017). This method is widely used in the detection of bacterial colony metabolic activity (Berry et al., 2015; Tao et al., 2017; Taubert et al., 2018; Olaniyi et al., 2019). The combination of D_2_O isotope labeling technology and single cell Raman spectroscopy can identify the Raman shift caused by the difference of metabolic activity between cells, which can be used as a semi-quantitative method to identify the metabolic activity of cells. Therefore, due to the pressure of antibiotics, differences in their metabolic activities lead to the displacement of C–D bonds between susceptible and resistant strains. The combination of the Raman and heavy water labeling techniques at the single-cell level can overcome the requirement of long-term culture in clinical pathogen experiments, making rapid drug screening possible.

In this study, we designed a single-cell Raman spectroscopy technique combined with a heavy water labeling as a rapid and accurate method for detecting antibiotic-resistant bacteria in respiratory bacteria. We established a Raman phenotype database of six common respiratory pathogens to provide a new method for rapidly identifying respiratory pathogens in clinical practice. We also explored different antibiotic-heavy water labeling conditions to achieve drug resistance identification of respiratory pathogens within 2 h. This study provides a rapid and sensitive method for identifying respiratory tract infection bacteria and their drug susceptibility.

## Methods

### Bacterial strains and culture conditions

We selected each clinical specimen, such as sputum and throat swabs, in the laboratory in our hospital from March 2020 to June 2020 to isolate *P. ae* and *MC*. The French VITEK2 COMPACT automatic bacteria analyzer and the M-H bacteria separation medium were used. The specimens were inoculated on the configured basic medium, placed in an ordinary incubator at a temperature of 35 °C and in an 8% CO_2_ incubator for 18–24 h, and then removed for bacterial identification. Approximately 0.9% normal saline was used to make pure colonies in the bacterial suspension 0.5 McDonnell unit, and the French VITEK2 COMPACT automatic bacterial analyzer was used to identify the bacterial species. Then, the results of Gram stain, colony morphology, and oxidase were carefully considered and combined to identify Acinetobacter baumannii, *Staphylococcus aureus, Pseudomonas aeruginosa* ATCC27853, and *Escherichia Coli* ATCC25922 as quality control strains.

### Antibiotic sensitivity assays

Bacterial pre-inoculums of the different strains were normalized to ~ 109 CFU/mL, and 10 μL of strains (~107 CFU/mL) was added with M-H basic medium. According to the Clinical and Laboratory Standards Institute (CLSI) guidelines, disk diffusion method was used to place different susceptibility disks on the medium containing amikacin, ceftazidime, ciprofloxacin, polymyxin E, ampicillin-sulbactam, cefepime, gentamicin, imimeropenem, piperacillin-ticarcillin, ticarcillin-clavulinic acid, tobramycin, welfare, piperacillin-tazobactam. The disks were incubated at 35 °C for 18–24 h, and the diameter of each inhibition zone was measured. The drug susceptibility results were judged according to the CLSI regulations, and the drug resistance was interpreted as susceptible (S), intermediate susceptible (IS), and resistant (R), following CLSI 2016 and EUCAST 6.0 (2016) guidelines and were compared (Weinstein, 2020).

### Heavy water labeling

The LB medium was mixed with heavy water concentrations of 10, 20, 30, 40, and 50%. Antibiotics were added into the culture at 0, 1, 2, 3, 6, and 12 h. The respiratory tract-resistant bacteria *Pseudomonas aeruginosa* and the sensitive bacteria *Escherichia Coli* were cultivated separately.

### Sample preparation and Raman spectral acquisitions

The bacterial pellet was washed two times with sterile water at 6,000 g for 5 min and resuspended to 1 mL. Approximately 3–5 μL of the sample was drop-cast onto a Raman chip and air-dried for 5–10 mins. Raman spectra were acquired from the dried bacterial drop using a Raman Microscope (HOOKE P300, HOOKE Instruments Ltd., China) equipped with a 532 nm laser. The data acquisition conditions were 600 gratings, the power on the samples was 5 mW, and the acquisition time was 5 s. Data from different amounts of individual cell Raman spectra were collected; 200 spectra for pathogen modeling data, 100 spectra for heavy water labeling, and 10 spectra for the clinical predicting model.

### Raman spectral data processing and analysis

#### Data preprocessing

Raman spectral data preprocessing is the premise of Raman qualitative analysis and quantitative analysis. It mainly applies Raman spectral smoothing based on the Savitzky–Golay filter, and Raman spectral baseline correction based on airPLS in HOOKE intP (HOOKE Instruments Ltd., China) software. The width of the Savitzky–Golay filtering window was set to 5 pixels, and third-order polynomial fitting was applied. The airPLS algorithm Lambda was 100, and the maximum number of iterations using itermaxAirPls was 15. All the resistant bacteria Raman data were batch processed with the same hyper-parameters.

#### Heat map analysis

Protein was represented by the 720, 785, 1,320, and 1,575 wave number peaks, nucleic acid by 1,004 cm^−1^ and 1,665 cm^−1^ peaks, and lipid by the 1,452 cm^−1^ peak in the distribution of *E. coli, K. pn, MC*, and the other six drug-resistant bacteria. The heat map was generated based on the linear background method which calculates the net peak area of 10 pixels around the wave number peak. The net peak area is shown in pseudocolor, where yellow is the weakest and red is the strongest signal.

#### Cluster analysis

The dimension of the Raman spectral data collected is 1,340 dimensions (CCD (charge-coupled device) camera pixel 1,340^*^100). Many of the 1,340 dimension features are noise or redundant information. In this study, the t-distributed stochastic neighbor embedding (t-SNE) algorithm was applied to dimensionality reduce the Raman data. t-SNE used t-distribution in low-dimensional space, which realized closer aggregation of points in the same cluster and further distance between points in different clusters. It effectively solved the problem of data point congestion in low-dimensional space, improving the visualization effect, and maintaining the data structure to a greater extent to achieve the expression of high-dimensional data in low-dimensional space. t-SNE was used for dimensionality reduction of the Raman data of six drug-resistant bacteria, and two-dimensional visualization analysis was carried out with t-SNE1 and t-SNE2, two maximum contribution dimensions. The results showed that the six drug-resistant bacteria were highly non-linear and showed non-homogeneity within clusters and dispersion between clusters.

#### Algorithm

A boosting integration strategy is adopted by the XGBoost algorithm, which is a boosting algorithm based on the decision tree. The gradient lifting algorithm is used to continuously reduce the loss of the previously generated decision tree and generate a new tree formation model, ensuring the reliability of the final decision. XGBoost performs second-order Taylor expansion on the loss function in the optimization process of gradient lifting decision tree (GBDT) and introduces second-order derivative information to make the model converge faster in the training process. In addition, XGBoost also adds regular terms to the loss function to control the model complexity and to prevent over-fitting.


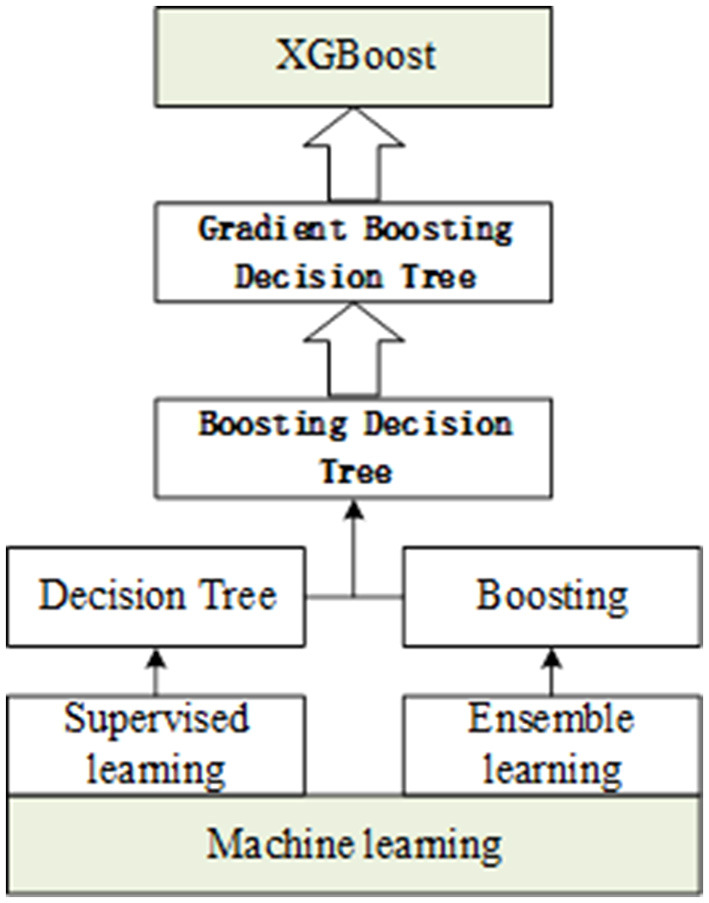


In this study, the XGBoost algorithm was applied to the recognition of “respiratory-resistant bacteria,” and the study was carried out from sample data preprocessing, feature extraction, and modeling analysis. The six kinds of spectral data were input into the XGBoost detection model, and a new decision tree was determined by iteratively learning the residual between the predicted value and the true value. The cumulative result of the tree gradually approaches the true value to complete the training, and then, the predicted probability of the classifier (A, B, C, D, E, and F; six substances) was used as the basis for discrimination. XGBoost was applied to identify them. First, the original spectral data were preprocessed to obtain the feature vector for model training; then, the model was optimized by grid search and 3-fold cross-validation; finally, the recognition model of “respiratory-resistant bacteria” was obtained. The importance score is a measure to evaluate the importance of each feature in the feature set to which it belongs. To improve the efficiency of generating new trees during the training process, XGBoost gives the importance score of each feature according to the gain value in each iteration, providing a basis for establishing a new tree with gradient direction in the next iteration. In this study, the importance score was used as the basis for quantifying the importance of each feature to select features and to extract the important features of the “respiratory pathogen” Raman spectrum.

### Cytotoxicity test

The medium containing 10, 20, 30, 40, and 50% heavy water was inoculated with *Pseudomonas aeruginosa* at a concentration of 2%. The culture was shaken at 200 rpm at 37 °C for 12 h. The pure culture medium was used to remove the background, and OD600 of the cultivated culture was detected using an ultraviolet spectrophotometer.

### Statistics

Comparisons between multiple groups were made by using a one-way analysis of variance and an appropriate posttest or a non-parametric equivalent, where appropriate. Analyses were performed using GraphPad Prism Software (San Diego, CA). All data are representative, and measurements were repeated at least three times. No data have been pooled.

### Study approval

All procedures were performed with approval from the ethics committees of the First Hospital of Jilin University.

## Results

### Single-cell Raman spectroscopy of respiratory tract pathogens

**Figure 1:**
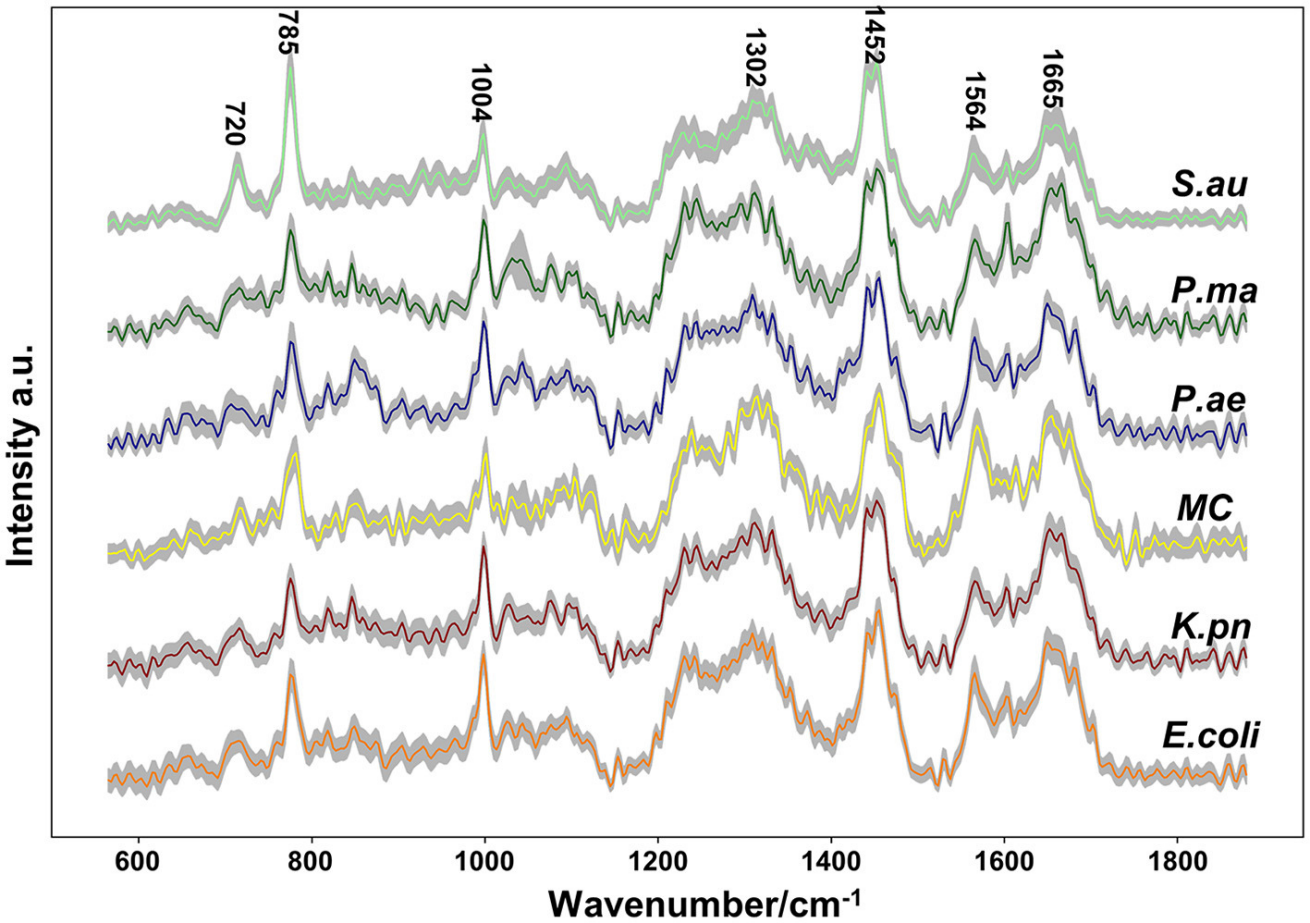
Average Raman spectra of six respiratory pathogens detected at an excitation wavelength of 532 nm. The solid line represents the spectral mean and the shadow represents the intra-group variance. The spectral band characteristics of each pathogen were displayed.

**Figure 2:**
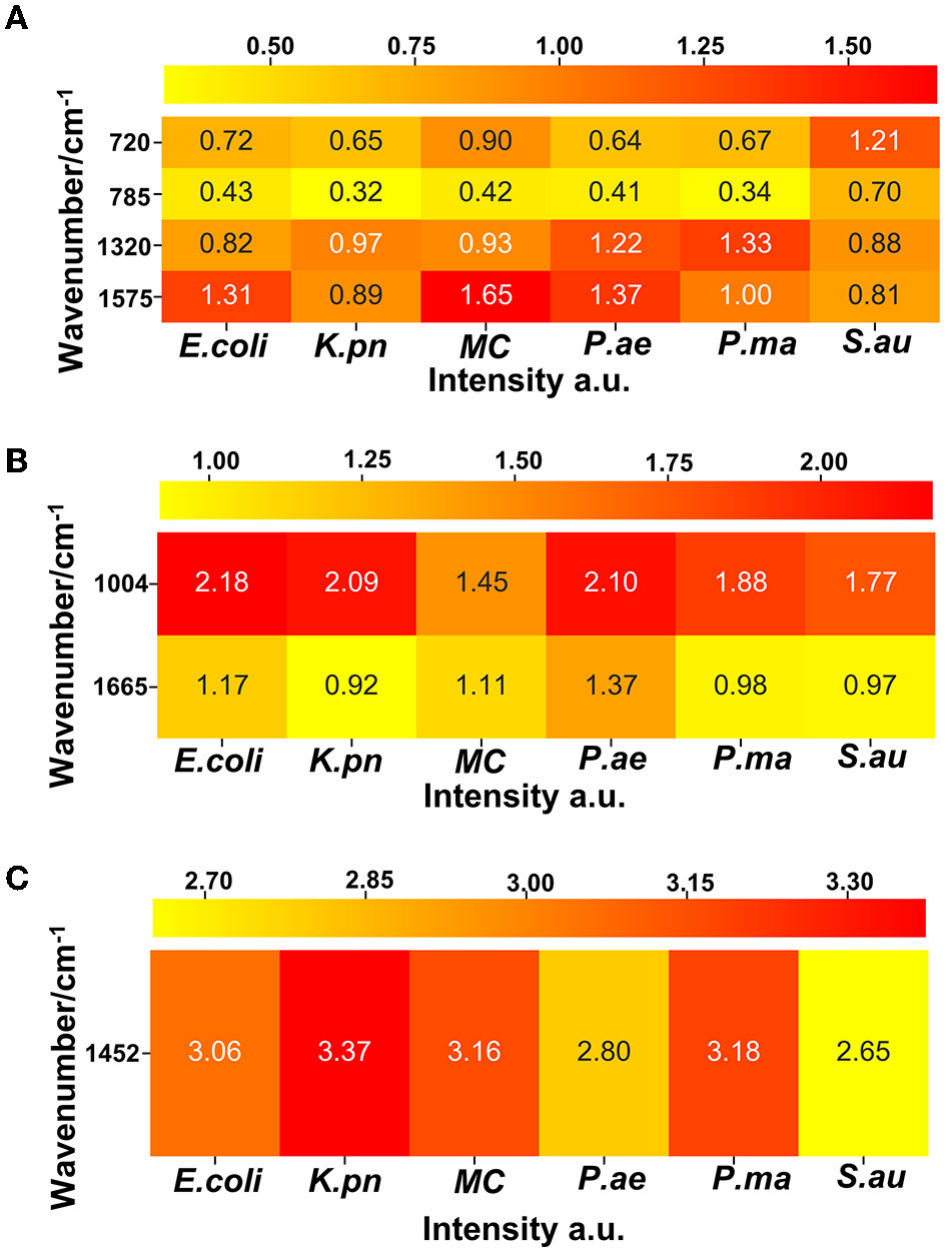
A heat map shows the intensity information of the characteristic peak bands of six respiratory pathogens, which can reflect the relative quantification of nucleic acids, proteins, and lipids corresponding to each pathogen. **(A)** Nucleic acid correlation peak, **(B)** protein correlation peak, and **(C)** lipid correlation peak.

Single-cell Raman spectroscopy is used to obtain microbial Raman spectra at the single-cell level, which can reflect the characteristic “fingerprint” of pathogens. Therefore, SCRS can analyze respiratory tract pathogens at the single-cell level. To analyze the physiological characteristics of common respiratory tract pathogens, we isolated, purified, and cultured six respiratory tract pathogens from clinical samples. The identification information of six respiratory pathogens is *Escherichia Coli* (*E. coli*), *Klebsiella Pneumonia* (*K. pn*), *Staphylococcus aureus* (*S. au*), *Moraxella Catarrhalis* (*MC*), *Ps. Melophilia* (*P. ma*), and *P. Aeruginosa* (*P. ae*). To establish a comprehensive and reliable training data set, 200 representative Raman spectra of the six strains were collected, and the characteristic spectra of each pathogen, obtained by computational analysis, are shown in Figure 1. The Raman spectrum fingerprint area (400–1,800 cm^−1^) represents the biochemical information of bacterial cells, including nucleic acids, proteins, and lipids. The Raman spectra of each bacterial species contain essential information for studying the complex structure of cells. Table 1 shows the significant differences in the peak positions of these six pathogens, mainly due to protein (1,004 and 1,665 cm^−1^), nucleic acid components (720, 785, 1320 cm^−1^), and lipid (1,452 cm^−1^) composition (Cardinali et al., 2019). The performance of the characteristic band of the Raman spectra of six kinds of microorganisms was analyzed to show the differences between these six kinds of microorganisms and nucleic acids, proteins, lipids, and other substances, as shown in Figure 2, according to the statistical analysis of the line chart and heat map.

**Table 1:** Raman bands in the spectra of childhood respiratory pathogens and their tentative assignments.

**Wavenumber/cm^−1^**	**Assignment**	**Reference**
720	Nucleic acids	(Cardinali et al., 2019)
785	Uracil	(James et al., 2008)
1,004	Phenylalanine	(Fendrihan et al., 2009)
1,320	Guanine	(Liu et al., 2008)
1,452	Lipids	(Lau et al., 2003)
1,575	Guanine	(Liu et al., 2008)
1,665	AmideI	(Lau et al., 2003)

Single-cell Raman spectroscopy of *E. coli* shows the most prominent Raman band at 1,004 cm^−1^, which is due to the in-plane rocking mode of –CH_3_ groups attached to the polyene chain (Fendrihan et al., 2009). The intensity of the 1,665 cm^−1^ band of *P. ae* is the strongest, and Amide I contributes to it (Lau et al., 2003). The intensity of the 1,452 cm^−1^ band of *K. pn* is the strongest, and the prominent band at 1,452 cm^−1^ is due to the –CH_2_ deformation mode of lipids (Fendrihan et al., 2009); the intensity of the 1,320 cm^−1^ band of *P. ma* is the strongest, the intensities of the 720 and 785 cm^−1^ bands of *S. au* are the strongest, and the 720, 785, and 1,320 cm^−1^ bands are due to nucleic acids (James et al., 2008; Liu et al., 2008). The spectra's peak positions and intensities are the pathogen's characteristic fingerprints (De Plano et al., 2019). The changes in these peak bands can be used to establish the Raman data model of pathogens.

Our study shows that Raman spectroscopy has excellent potential to analyze physiological characteristics and can deeply analyze the vibration information of different molecules. The unique and distinguishable Raman bands, with multiple biomarkers in SCRS, allow the profiling of respiratory pathogens at the single-cell level.

### Differential differentiation and classification prediction model of respiratory pathogens

**Figure 3:**
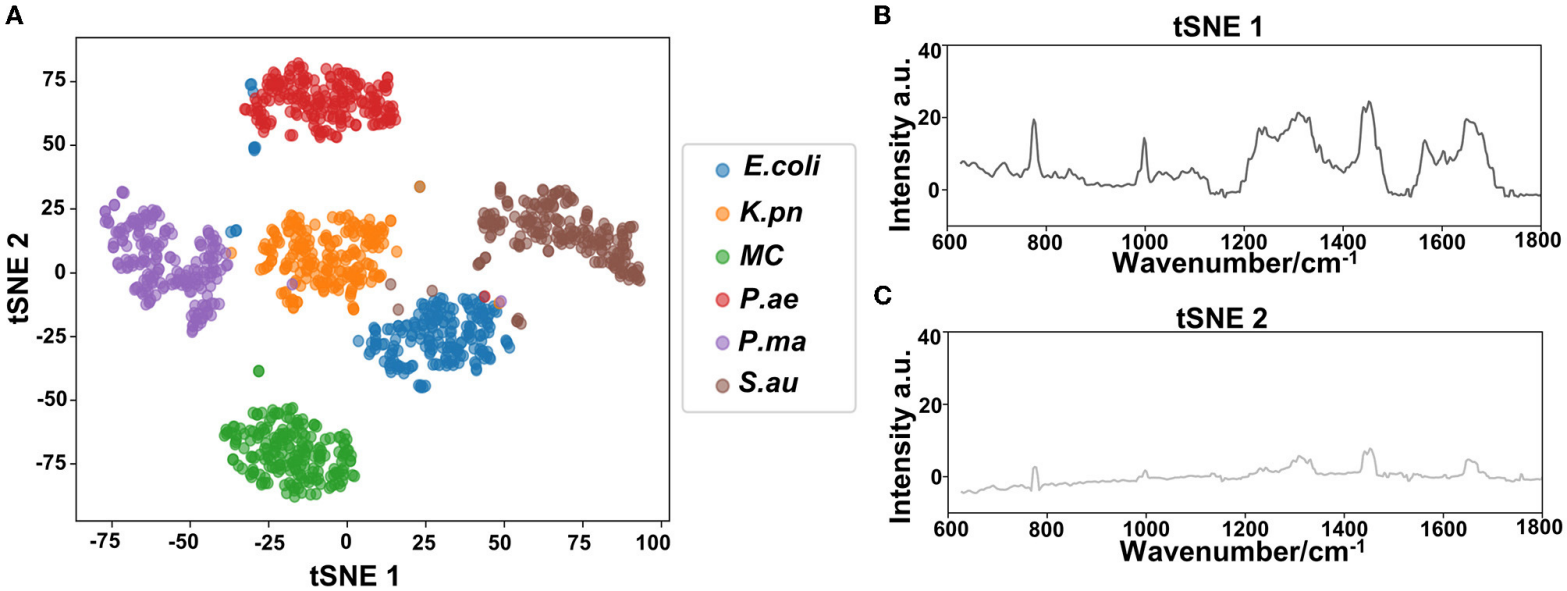
Classification analysis of six respiratory pathogens. **(A)** t-SNE visualization analysis and **(B, C)** Raman wavenumber loading plots against the contributions of relevant Raman bands to t-SNE1 and t-SNE2 of the t-SNE.

**Figure 4:**
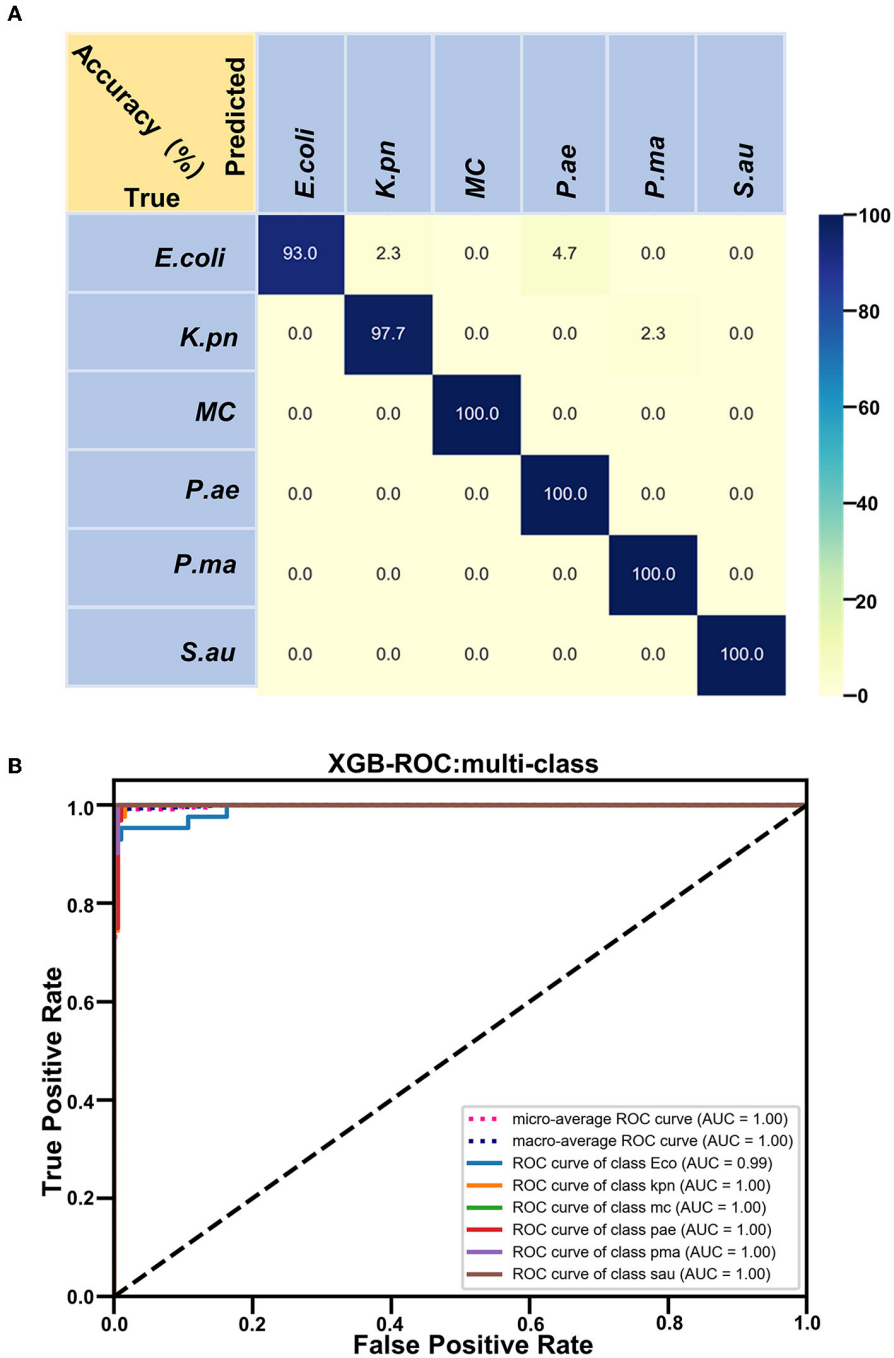
**(A)** XGB modeling and prediction results. **(B)** XGB analysis of ROC curve.

Based on the Raman spectra of these macromolecules and combined with data analysis, structural information of biological macromolecules can be obtained. At present, Raman spectroscopy can be used to identify strains of species that have been confirmed (Maquelin et al., 2003). t-SNE is a machine learning method for dimensionality reduction, which can help us identify associated patterns (Pezzotti et al., 2017). The main advantage of t-SNE is the ability to maintain local structure. This means that points with close distances in a high-dimensional data space are projected to be close in a low-dimensional data space. This method can make points in the same cluster gather more closely, and points in different clusters move farther apart, effectively solving the problem of crowded data points in low-dimensional space (Zhou and Jin, 2020). First, 1200 spectral datasets from six pathogens were analyzed by the t-SNE algorithm. The differences among the six pathogenic bacteria can be obtained from the results of Raman spectra in the early stage, and the t-SNE results could further demonstrate the differences among the six pathogenic bacteria. Each bacterium was completely clustered into the same cluster species, and different bacteria were clustered into different clusters, which are divided into six clusters. Therefore, the spectral differences between the six bacteria were significant, which can realize the characteristic differences between species, as shown in Figure 3A. The first two feature dimensions of t-SNE were reconstructed after dimensionality reduction, as shown in Figures 3B, C, which show Raman wavenumber loading plots against the contributions of relevant Raman bands to t-SNE1 and t-SNE2 of the t-SNE. XGBoost is a classical integrated lifting algorithm framework with high training efficiency, good prediction effect, controllable parameters, ease of use, and other characteristics; it is a sharp tool in the field of extensive data analysis and is widely used in biomedicine, environmental detection, and other fields, achieving excellent results. XGBoost is used to reveal complex information in Raman spectra and to identify and classify bacteria in biological studies. In this study, we trained the classification model, modeled six respiratory pathogens, and evaluated the predictive ability of the XGBoost model using the entanglement validation method. The results shown in Figure 4A show that XGBoost has a very accurate prediction effect on the six respiratory pathogens, with an overall prediction accuracy of 93–100%. The lowest prediction sample is *E. coli*, with an accuracy of 93%, and *MC, P. ae, P. ma*, and *S. au* all reach 100%. The ROC curve was applied to evaluate the sensitivity and specificity of the model, as shown in Figure 4B. The ROC of the label dimension and sample dimension rapidly approached the upper left corner, and the sum of the sensitivity (TPR) and specificity (FPR), expressed by the calculated AUC value, was close to 1, indicating that the model was adequate and had a good performance.

In our study, six respiratory pathogens have apparent differences from the results of the two clustering and classification algorithms. According to t-SNE1 and t-SNE2, six clusters are clustered, and the classification accuracy of the machine learning model is more than 93%, which provides a prediction model for diagnosing clinical respiratory pathogens.

### Raman combined with heavy water labeling to detect the drug resistance of *Pseudomonas aeruginosa*

**Figure 5:**
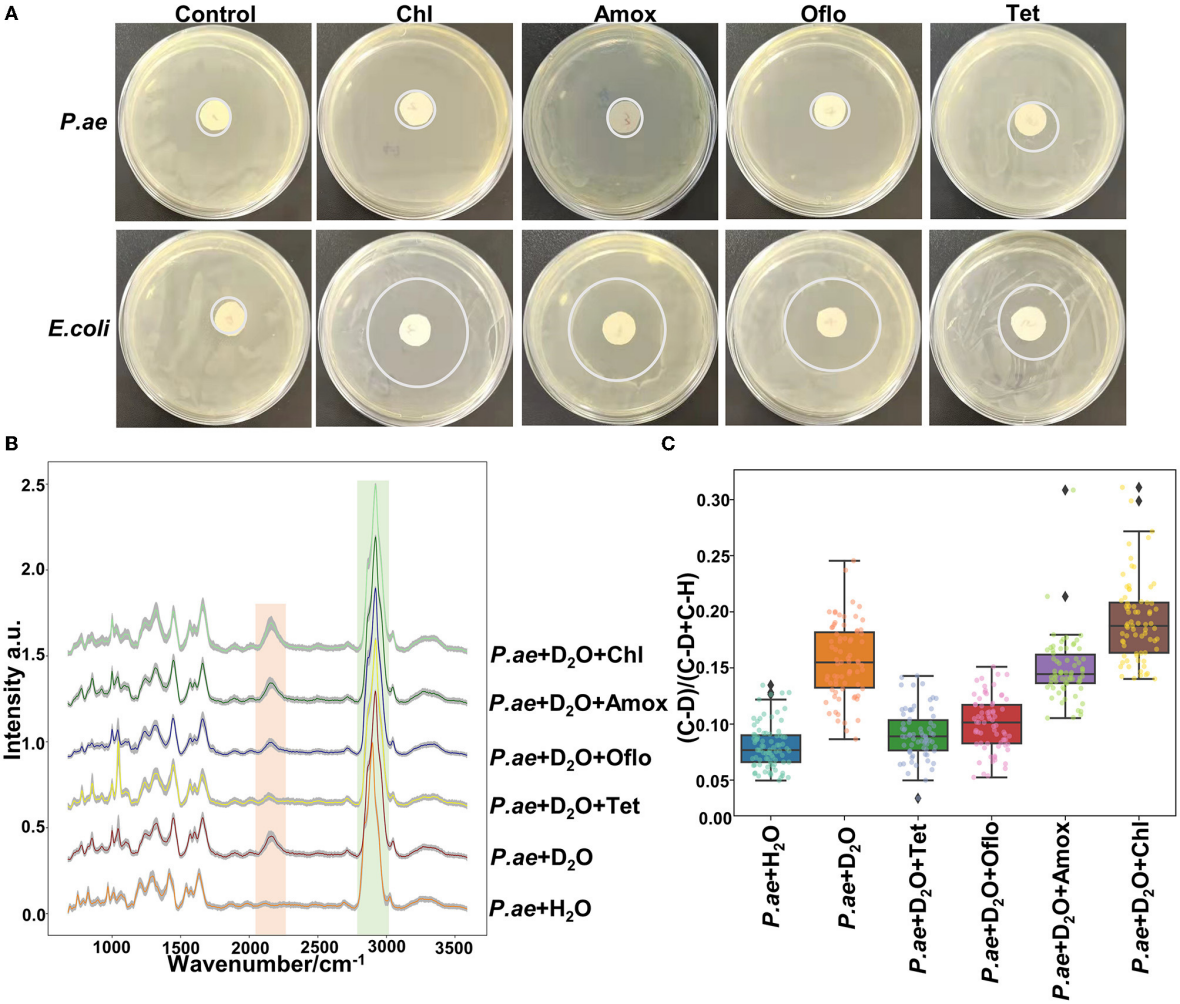
Resistance of *Pseudomonas aeruginosa* to different antibiotics. **(A)** Representative images of *Pseudomonas aeruginosa* and *E. coli* in different antibiotics. **(B)** Spectra of *Pseudomonas aeruginosa* in different antibiotics. **(C)** C–D ratio analyzed by heavy water labeling. The C–D peak was located at 2,040–2,300 cm^−1^ and the C–H peak at 2,800–3,100 cm^−1^. According to C–D/(C–D + C–H), the activity of drug-resistant bacteria was determined. The antibiotics used were cefazolin, amoxicillin, tetracycline, and ofloxacin.

Metabolically active microorganisms metabolize deuterium into the cell through the NADH/NADHP-oxidized respiratory chain, and heavy water is added to the newly synthesized protein, lipid, and DNA (Hekmatara et al., 2021). Deuterium forms a chemical bond with carbon, causing the C–H peak to shift, and a new C–D peak appears. The shift speed can reflect the synthesis speed of cell macromolecules, and the changes in their metabolic activity can be characterized for the same kind of cells. The drug resistance of respiratory pathogens was evaluated according to the C–D (2,040–2,300 cm^−1^) and C–H bands (2,800–3,100 cm^−1^) by Raman spectroscopy and the metabolic activity of the cell was also evaluated by calculating the ratio of the peak area in the C–D/(C–D + C–H) spectrum (Yi et al., 2021). As the drug resistance detection sample, we selected a representative strain of drug-resistant *Pseudomonas aeruginosa* from six respiratory pathogens. We selected a standard strain of non-resistant *Escherichia Coli* as the control group. Cefazolin, amoxicillin, ofloxacin, and tetracycline were selected for the experiment. As shown in Figure 5A, the results of the bacteriostatic zone experiment showed that *Pseudomonas aeruginosa* was resistant to all four antibiotics. In comparison, non-resistant *Escherichia Coli* was not resistant to all four antibiotics. Table 2 shows the diameter of the bacteriostatic circle. Then, *Pseudomonas aeruginosa* was labeled with heavy water and 30% heavy water and corresponding antibiotics were added to the medium without heavy water culture as the set control group. After culturing for 12 h, the samples were collected for Raman analysis. The results showed that the C–D peak was detected in all antibiotic treatment groups, indicating that *Pseudomonas aeruginosa* had different degrees of resistance to the four antibiotics, as shown in Figure 5B. Furthermore, the C–D rate in the various culture treatments is shown in Figure 5C. Therefore, we conclude that the drug resistance of respiratory pathogens can be detected by heavy water labeling combined with Raman technology.

**Table 2:** Diameter of the bacteriostatic circle in different antibiotics.

**Class of antibiotics**	**Inhibitory Zone Diameter (IZD)**	
	***E.coli***	***P.ae***
Chloramphenicol	30 ± 2 mm	10 ± 3 mm
Amoxicillin	27 ± 4 mm	10 ± 1.5 mm
Ofloxacin	25 ± 1 mm	10 ± 2 mm
Tetracycline	22 ± 2 mm	12 ± 1 mm

### Rapid detection of resistance of *Pseudomonas aeruginosa* to Amoxicillin

**Figure 6:**
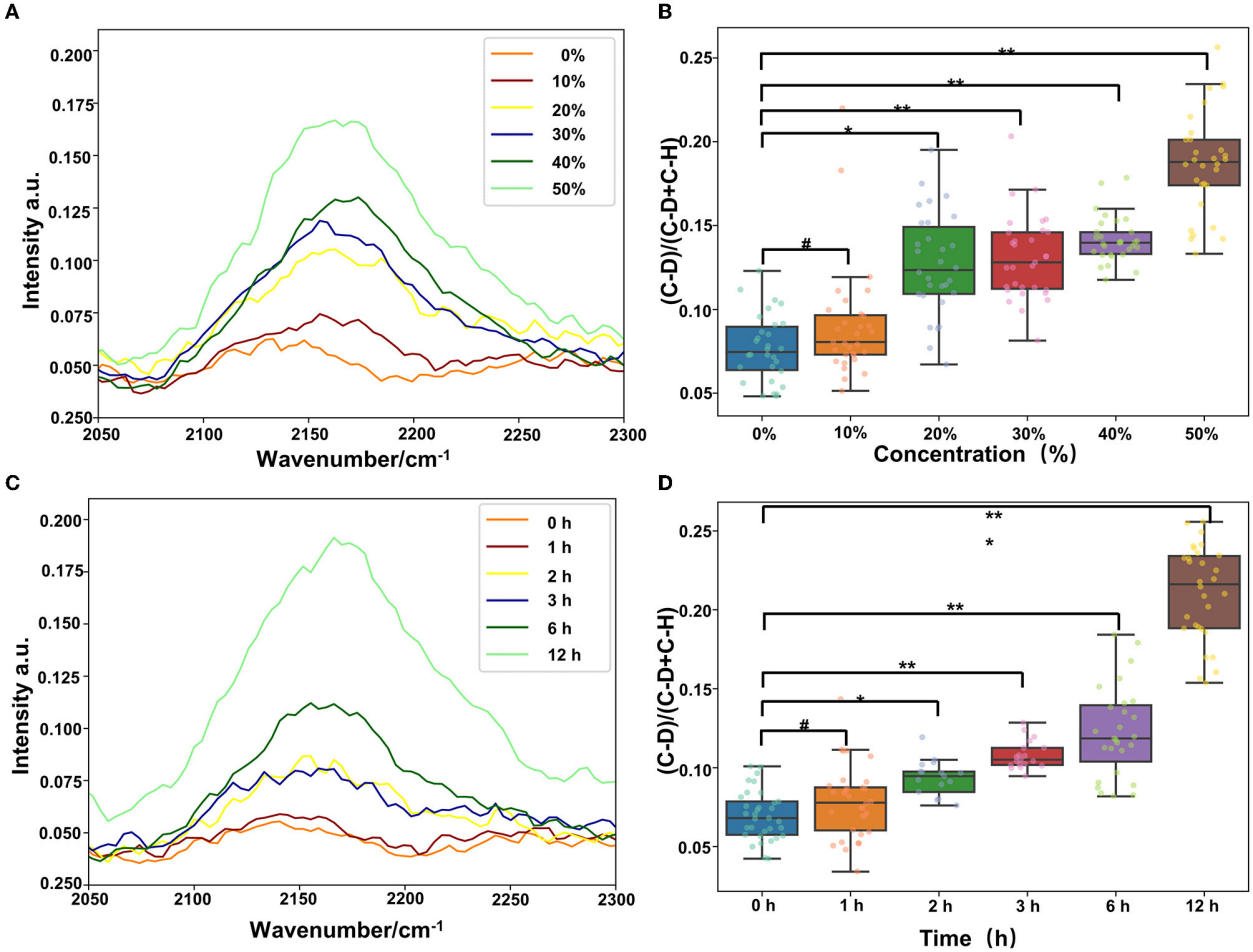
Heavy water can quickly mark the activity of bacteria, and, combined with Raman spectroscopy, we can quickly detect the drug resistance (^#^*p* > 0.05; *p < 0.05; **p < 0.01; and ***p < 0.001). **(A)** C–D peak location map of *Pseudomonas aeruginosa* labeled with different heavy water concentrations. **(B)** Statistics of the relative peak intensity map of C–D labeled with different heavy water concentrations. **(C)** C–D peak location map of *Pseudomonas aeruginosa* labeled with different heavy water labeling time and **(D)** statistics of the relative peak intensity of C–D labeled with different heavy water labeling time.

The clinical detection cycle of drug-resistant bacteria is normally 2–3 days. To save time and to provide advice to clinicians for the infected bacteria and sensitive antibiotics, we found that the combination of Raman technology and heavy water marking can realize the detection of drug-resistant bacteria. Therefore, we set different concentrations of heavy water (10, 20, 30, 40, and 50%) to label *Pseudomonas aeruginosa*. As shown in Figure 6, a 20% heavy water concentration can achieve the labeling. The abundance of the labeling becomes higher as the concentration increases (Figures 6A, B). Then, we selected the 30% heavy water concentration to evaluate the time that could be marked (0, 1, 2, 3, 6, and 12 h). The results in Figures 6C, D show that 2 h could be marked, but 3 h was better. Therefore, it is recommended that the marking time should be 3 h. This can significantly shorten the identification and detection time of the drug resistance of clinical pathogens.

Our study determined conditions for labeling *Pseudomonas aeruginosa* with heavy water. The combined application of Raman and heavy water labeling technologies can realize the rapid resistance of respiratory tract pathogens within 2 h, which provides a new feasible scheme for the rapid detection of respiratory tract pathogens.

### Evaluation of the effect of heavy water on cell activity

**Figure 7:**
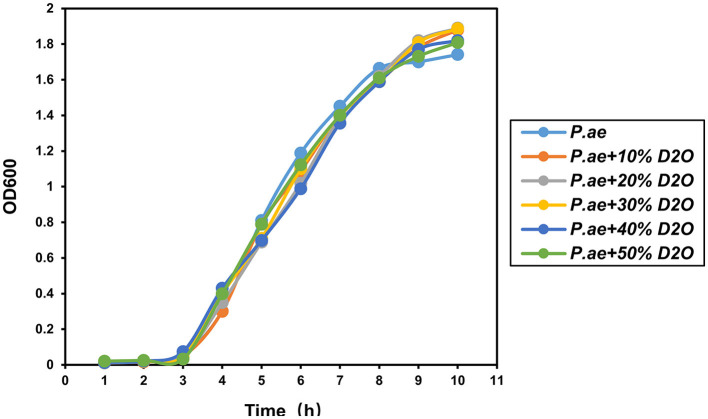
Effect of heavy water on cell viability. Experiments showed that it did not affect the proliferation and viability of bacteria.

The results of previous experiments showed that using heavy water combined with Raman can rapidly detect pathogen resistance within 2 h. Testing whether the heavy water is toxic to the cells is also necessary. Therefore, this evaluation was carried out by culturing different concentrations of heavy water for 12 h. The results showed that heavy water was not toxic to the bacteria during the culture process and did not affect cell activity. It is a non-toxic and rapid-labeling reagent, as shown in Figure 7.

### Identification of samples from patients with clinical respiratory tract infection

**Figure 8:**
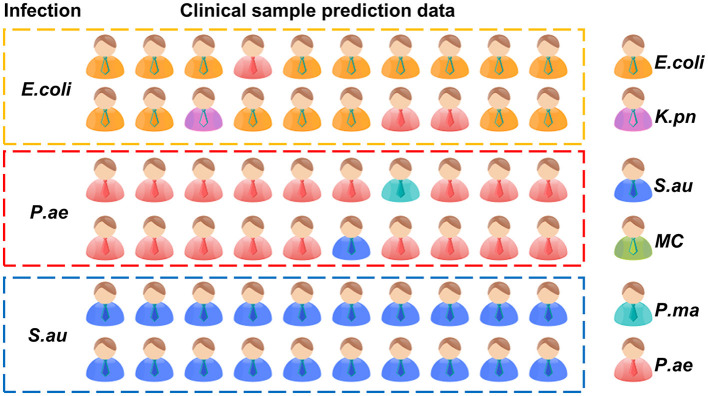
XGB model established to predict the pathogen in clinical samples.

To verify the rapid Raman detection in new clinical samples, we collected six samples from clinically infected patients and isolated them, which were clinically identified as two strains of *E. coli*, two strains of *P. ae*, and two strains of *S. au*. A total of 10 single cells were randomly selected from each sample for Raman spectroscopy analysis. The XGB model established above was used to predict clinically separated samples. The prediction results of *E. coli* showed that there were three data discriminated as *P. ae*, one data discriminated as *K. pn*, and the prediction accuracy rate was 80%. The *P. ae* forecast result showed that one of the data was discriminated as *P. ma* and the other as *K. pn*, and the statistical prediction accuracy was 90%. *S. au* prediction error was 0, and the prediction accuracy rate was 100%. The result is shown in Figure 8. Infectious diseases are common clinical diseases. Rapid and accurate diagnosis is key to infection control (Domenech et al., 2018). The identification method of clinical pathogens relies on the traditional bacterial culture, isolation, purification, and identification, which is cumbersome, has a long detection cycle, and cannot timely and effectively guide the application and treatment of clinical antibiotics (Leski et al., 2011). Our experimental results showed that it is possible to collect only 10 Raman spectra of microorganisms per patient for prediction and discrimination, and then reduce the culture of pathogens. The clinical testing time is considerably shortened. At the same time, the discriminant error between *E. coli* and *P. ae* shows that the training model has the potential for continuous improvement.

The prediction accuracy of the XGB respiratory pathogen model was 80% for *Escherichia coli*, 90% for *Pseudomonas aeruginosa*, and 100% for *Staphylococcus aureus*.

## Discussion

This study aimed to explore a rapid and accurate method of identifying various susceptible or antibiotic-resistant bacterial strains from patients with respiratory infections. Early recognition of antibiotic-resistant strains would effectively guide the choice of antibiotics in the clinic and would avoid the problem of antibiotic resistance. Respiratory tract infections, particularly lower respiratory tract infections (LRTI), are the leading global causes of morbidity and mortality (Roth et al., 2018). Our study on antibiotic susceptibility respiratory bacteria testing would greatly help in prescribing antibiotics.

Through our assays, it is critical to determine the remarkable bio-molecular profiling of the bacteria at the excitation wavelength of 532 nm, which is suitable for cytological samples on glass substrates (Kerr et al., 2015). Nucleic acids, uracil, phenylalanine, CH_2_ bending mode of lipids, and amide I were separately observed in the Raman spectra at 720, 785, 1,004, 1,452, 1,575, and 1,665 cm^−1^, consistent with previous literature. Furthermore, guanine presented at both 1,320 and 1,575 cm^−1^. Through the Raman intensity of nucleic acids, proteins, and lipids in each respiratory pathogen, we could identify *E. coli, K. pn, S. au, MC, P. ma*, and *P. ae*, listed in the heat map in Figure 2.

To improve the accuracy of the identification of each pathogen, we used several calculation methods. We found that the combination of t-SNE cluster analysis and the XGB algorithm was the best choice for data processing and analysis. According to our algorithm, the Raman spectra of each respiratory pathogen were gathered into six clusters. These representative data were used to distinguish the six kinds of pathogens with an accuracy of 100% in four kinds of pathogenic bacteria. Furthermore, the lowest accuracy of the other pathogenic bacteria was 93%. The peak positions of each respiratory pathogen could be used as the marker of the common bacteria. At the same time, the data should be completed to represent the majority of the respiratory pathogens.

To resolve the problems with identifying bacteria, we aimed to provide the drug resistance of each bacteria to acquire more information about the pathogens. By deuterium attaching to the metabolically active microorganisms, the C–D bond reflected the metabolic activity of the bacteria. The exposure to antibiotics inhibited the proliferation of sensitive bacteria and resulted in common spectral changes such as the C–D drift in the Raman intensity. *Pseudomonas aeruginosa* was treated with cefazolin and amoxicillin, in heavy water and mixed medium, respectively, and the C–D bond ratio was detected. As cefazolin and amoxicillin belong to beta-lactam antibiotics and this cell wall–targeting antibiotic causes DNA damage (Maiques et al., 2006), the Raman spectral changes are likely to be similar for both classes. Tetracycline antibiotics bind to the 30S subunit of ribosome at the mean, while those of ofloxacin antibiotics inhibit the activity of bacterial DNA helicase. The Raman spectral changes with different antibiotics are diverse. However, the C–D bond ratio was significant in comparing and contrasting the spectral changes with or without antibiotics. The C–D peak was located at 2,040–2,300 cm^−1^ and the C–H peak at 2,800–3,100 cm^−1^.

As a hypothesis, *Pseudomonas aeruginosa*, a naturally resistant strain to the first and second-generation cephalosporins and amoxicillin, had different degrees of resistance to cefazolin and amoxicillin. *Pseudomonas aeruginosa* exposed to tetracycline antibiotics showed a similar C–D ratio to the ofloxacin antibiotics. It was believed that tetracycline antibiotics were less susceptible to *Pseudomonas aeruginosa* (Grossman, 2016). Further studies with different mechanisms of antibiotics and bacterial species should be performed to confirm the true potential of the C–D ratio in assessing the antibiotic susceptibilities of bacteria resistance. It was also significant to confirm the exposure duration of bacteria to the antibiotics and the concentrations of heavy water. The shortest time for confirming *Pseudomonas aeruginosa* drug susceptibility, during which Raman spectroscopy detected the C–D drift, was 3 h. In comparison, the current clinical protocol for isolating bacteria takes 24 h to 5 days before AST.

In summary, our study demonstrates the potential of 20% D_2_O labeling Raman spectroscopy in confirming the resistance of different antibiotics to respiratory bacteria within 3 h, without time-consuming, complex, or tedious processing. Understanding how antibiotics influence bacterial metabolism may result in the development of better therapeutic strategies and may help to avoid the production of resistant bacteria. In the future, it is also necessary to correlate the Raman spectral data to the AST results and to track the capacity of bacteria converted to resistant phenotypes after the use of antibiotics in the clinic.

## Conclusion

In this study, SCRS and t-SNE analysis algorithms were used to visualize the significant differences among six respiratory tract pathogens, which could be significantly isolated. Based on the heat map analysis, each bacterium has a characteristics in peak intensity, which may be used as the basis for classification and differential analysis. The XGBoost machine learning classification model was used to classify six respiratory tract pathogens with an accuracy of 93–100%. The classification accuracy of patients with clinical respiratory tract infections was more than 80%. Therefore, it is expected that the SCRS technology can be used to rapidly identify respiratory pathogens in non-culture conditions. The Raman technique combined with heavy water labeling technology detected the drug resistance of respiratory pathogens. It was determined that the drug resistance could be identified within 2 h. Heavy water did not affect microbial activity. Therefore, our study provides a new feasible scheme for the identification and drug resistance detection of respiratory pathogens using SCRS–D_2_O.

## Data availability statement

The original contributions presented in the study are included in the article/supplementary material, further inquiries can be directed to the corresponding author.

## Author contributions

YZ and ZL contributed to conception and design of the study. YX performed the statistical analysis. CY wrote the first draft of the manuscript. BL wrote sections of the manuscript. All authors contributed to manuscript revision, read, and approved the submitted version.
